# Radiation-Induced Intestinal Injury: Injury Mechanism and Potential Treatment Strategies

**DOI:** 10.3390/toxics11121011

**Published:** 2023-12-10

**Authors:** Qianying Lu, Yangfan Liang, Sijia Tian, Jie Jin, Yanmei Zhao, Haojun Fan

**Affiliations:** 1 Institute of Disaster and Emergency Medicine, Tianjin University, Tianjin 300072, China; qianying.lu@tju.edu.cn (Q.L.); liangyangfan@tju.edu.cn (Y.L.); a3373348309@163.com (S.T.); jinjie1998@tju.edu.cn (J.J.); 2 Tianjin Key Laboratory of Disaster Medicine Technology, Tianjin 300072, China

**Keywords:** radiation, intestinal injury, injury mechanism, treatment

## Abstract

Radiation-induced intestinal injury (RIII) is one of the most common intestinal complications caused by radiotherapy for pelvic and abdominal tumors and it seriously affects the quality of life of patients. However, the treatment of acute RIII is essentially symptomatic and nutritional support treatment and an ideal means of prevention and treatment is lacking. Researchers have conducted studies at the cellular and animal levels and found that some chemical or biological agents have good therapeutic effects on RIII and may be used as potential candidates for clinical treatment. This article reviews the injury mechanism and potential treatment strategies based on cellular and animal experiments to provide new ideas for the diagnosis and treatment of RIII in clinical settings.

## 1. Introduction

Cancer threatens people’s physical and mental health. The World Health Organization (WHO) reports that about 8.8 million people died of cancer worldwide in 2015 [1]. A study shows that by 2023, the United States is expected to have 1,958,310 new cancer cases and 609,820 cancer deaths [2]. Therefore, finding methods to reduce the death rate of cancer is one of the most important goals of modern society in order to preserve human health. Radiation therapy is an effective way to treat malignant tumors and is used in more than half of all cancer patients [3]. Although radiation therapy brings benefits to cancer patients, its side effects on normal tissues cannot be ignored.

Ionizing radiation acts on the body and can cause direct and indirect damage. On the one hand, ionizing radiation directly transfers energy to the body’s biological macromolecules, such as DNA, RNA, and proteins. On the other hand, it ionizes water and produces free radicals, such as superoxide anions (O_2_^−^), hydrogen peroxide (H_2_O_2_), and hydroxyl radicals (OH^−^), which interact with biological macromolecules and cause damage to the body [4,5]. The small intestine renews rapidly and is highly sensitive to radiation. When the entire intestinal tract receives radiation doses exceeding 5 Gy, gastrointestinal injury occurs [6]. Accidental radiation exposure or clinically planned radiation therapy for abdominal/pelvic tumors can easily injure the intestine, resulting in radiation-induced intestinal injury (RIII). As a result, 50% of patients who have undergone abdominal/pelvic radiation therapy experience varying degrees of chronic intestinal dysfunction, leading to side effects that include vomiting, weight loss, anorexia, dehydration, diarrhea, and infections [3]. In severe cases, it can lead to intestinal necrosis, intestinal perforation, and even death [7,8]. Thus, RIII is one of the most common side effects among long-term cancer survivors and it has a significant impact on the quality of life of patients. 

However, the treatment of RIII is basically symptomatic and nutritional support treatment, such as anti-diarrhea, anti-vomiting, anti-inflammation, and surgical treatment, and an ideal means of prevention and treatment is lacking [9,10]. At present, researchers have conducted studies at the cellular and animal levels and found that some chemical or biological agents have good therapeutic effects on RIII and may be used as potential candidates for clinical treatment. In this article, we aimed to summarize the pathogenesis and potential therapeutic strategies of RIII and provide new ideas for the diagnosis and treatment of RIII.

## 2. Injury Mechanism of Radiation-Induced Intestinal Injury

The intestines coil in the abdomen and pelvis and, due to their large surface area, they cannot be shielded, so accidental radiation exposure or radiation therapy for abdominal/pelvic tumors will inevitably irradiate the intestines, which may cause enterotoxicity. Intestinal toxicity in patients with abdominal and pelvic tumors following radiotherapy correlates with small bowel dose parameters. Yao et al. [11] showed that V20 (the absolute volume of a small bowel receiving more than 20 Gy) and V55 independently influenced the occurrence of grade ≥2 acute RIII and that V20 = 183 cm^3^ and V55 = 2 cm^3^ were radiologically tolerable doses for the small bowel. Estabrook et al. [12] demonstrated that a critical dose of grade 3 or higher toxicity occurred when an area of 120 cm^2^ was irradiated with 15 Gy or an area of 195 cm^2^ was irradiated with 45 Gy. Bae et al. [13] found that V25 > 20 cc, with maximum point doses of 35 Gy and 38 Gy (more than three times), predicted severe gastroduodenal toxicity.

**Figure 1:**
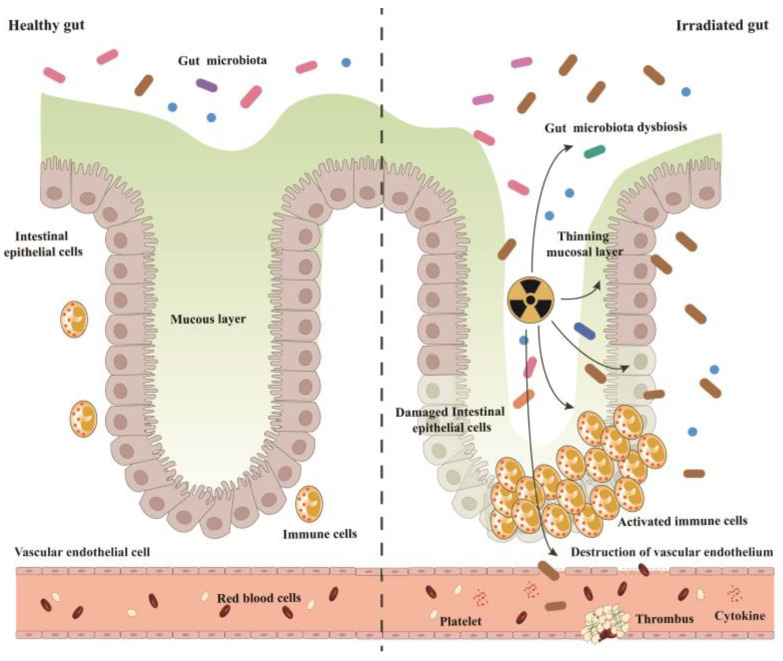
Healthy and irradiated gut. Left: Healthy gut. The gut consists of gut microbiota, the mucous layer, intestinal epithelial cells, immune cells, and vascular endothelial cells. Right: irradiated gut. After irradiation, gut microbiota dysbiosis, mucosal layer thinning, intestinal epithelial barrier breakdown, immune cell activation, and vascular endothelium destruction occurred.

Excessive ionizing radiation exposure can lead to the destruction of biological macromolecules, which causes damage to the intestinal epithelial barrier and intestinal vascular endothelial cells and affect intestinal immunity and intestinal microorganisms (Figure 1), resulting in local inflammation, mucosal edema, bleeding and coagulation disorders, bacterial infection, and even death.

### 2.1. Intestinal Epithelial Barrier Damage

The intestinal mucosal epithelial cells, together with the basement membrane, constitute the intestinal epithelial mechanical barrier. The intestinal epithelium comprises a large number of repetitive, continuously self-renewing crypt-villus units [14]. Intestinal stem cells (ISCs) in crypts are considered the source of all intestinal cells. They are differentiated into different functional cells during villus axis migration. Stem cells divide and produce transit amplifying cells (TA cells), which then differentiate downward into Paneth cells and upward into goblet cells, enteroendocrine cells, and intestinal absorptive cells [15]. Intestinal epithelial cells are held together by tight junction (TJ) proteins, which serve as a basic line of defense against potential intestinal pathogens [16]. However, the epithelium of the small intestine renews very quickly and is extremely sensitive to radiation, which disrupts its integrity [17,18]. This promotes water, electrolyte, and protein leakage into the intestinal cavity, and it also increases the chances of contact between the intestinal epithelium and pathogenic microorganisms, which aggravates the inflammatory response of the intestinal tract, leading to sepsis and death [19]. In the small intestine after injury, epithelial loss, fewer crypts, shortening of the villi, higher bacterial colony counts in mesenteric lymph nodes (MLNs), and higher FITC-dextran concentrations in the sera and bacterial 16S in the liver are observed, which indicates gut mechanical barrier disruption and intestinal dysfunction [20,21,22].

The structural basis of the intestinal mechanical barrier is the normal architecture of the intestinal mucosal epithelial cells and the tight junctions between the cells mediated by claudins, zonula occludens-1 (ZO-1), and catenin [23]. Radiation can cause DNA damage to intestinal epithelial cells via direct or indirect means, leading to cell death and tight junction damage, which subsequently disrupt the integrity of the intestinal mechanical barrier [24]. 

After exposure to ionizing radiation, the main death mode of intestinal epithelial cells is apoptosis. Studies have shown that p53 plays an important role in radiation response after intestinal injury. Ionizing radiation can cause DNA damage, which subsequently causes phosphorylation, activation, and accumulation of the p53 protein, thereby initiating p53-dependent apoptosis by activating downstream P53 up-regulated modulator of apoptosis (PUMA), NOXA, tumor necrosis factor (TNF), and factor-related apoptosis (FAS) [25]. Indeed, in an RIII model, the apoptosis of mouse intestinal cells and the expression of p53 were increased significantly. Pharmacologically blocking p53-dependent apoptosis by targeting PUMA or NOXA significantly protects intestinal damage from radiation [26,27]. In addition to apoptosis, recent studies have shown that autophagy, pyroptosis, and ferroptosis are also involved in radiation-induced intestinal epithelial injury. For example, Qu et al. [28] reported that IR increased the abundance of autophagy-related genes in the colonic mucosa of mice. Additionally, an autophagy inhibitor or intrinsic autophagy deficiency induces oxidative stress and inflammatory responses in intestinal epithelial cells, thereby improving intestinal barrier dysfunction [28,29]. Pyroptosis is a type of programmed cell death that is activated by inflammasome [30], and ferroptosis is an iron-dependent form of regulatory cell death [31]. Kiang et al. [32] reported that mixed-field (67% neutron + 33% gamma) radiation significantly increased active Gasdermin D, a biomarker of pyroptosis. It was reported that Gasdermin D was activated by caspase-1 and was increased in the ileum of mice after radiation. Zhang et al. [33] reported that in RIII, the levels of pyroptosis-related inflammatory factors, such as interleukin 1β (IL-1β), IL-18, and caspase-1 activity, as well as ferroptosis-related proteins, such as glutathione peroxidase 4 (GPX4) and 4-hydroxynonenal (4-HNE), were significantly increased. These studies indicate the role of pyroptosis and ferroptosis in RIII. The death of intestinal epithelial cells leads to intestinal epithelial damage. As mentioned above, ISCs are the source of all intestinal cells and are responsible for epithelial regeneration [16]. If ISCs are functioning properly, they proliferate rapidly to repopulate multiple adjacent crypts and villi after injury [34]. However, if ISCs are damaged, on the one hand, the missing cells cannot be replenished in time, which leads to the destruction of intestinal integrity. On the other hand, reduced proliferative activity of the ISCs leads to shortened villi, which will affect intestinal function. The intestinal villi comprise most of the surface of the intestine, which is essential for effective absorption [17]. The length of the intestinal villi is determined by the balance between epithelial cell proliferation and mortality [35]. Therefore, when ISCs are damaged, the proliferation of epithelial cells cannot supplement the death of epithelial cells in time, resulting in shortened villi, which subsequently reduces the intestinal surface area and intestinal absorption function.

Tight junction molecules consist of occludin, claudins, and junction adhesion molecules (JAMs), and they regulate the paracellular permeability of water, ions, and macromolecules in adjacent cells [36]. Once the tight junctions of intestinal epithelial cells are mutated, reduced, or lost, the permeability of the intercellular space will increase, and bacteria, endotoxins, and macromolecules can enter the systemic circulation [37]. Researchers have found that the levels of claudin-2, claudin-3, claudin-4, claudin-10, JAM-1, and occludin are changed in the radiation group, which disturbs the epithelial barrier and increases intestinal permeability [20,38]. Shukla et al. [39] reported that after mice were subjected to low-dose radiation (4 Gy), the redistribution of both occludin and ZO-1 from the intercellular junctions in the intracellular compartment and the loss of the junctional distribution of Cldn-3 in both the colon and ileum occurred. The abovementioned research shows that damage to tight junctions is one of the most important mechanisms of RIII.

### 2.2. Vascular Endothelial Damage

There are abundant capillaries in the villi of the small intestine, which transport nutrients throughout the body and oxygen to the small intestine. Capillaries comprise only one layer of vascular endothelial cells, which are exquisitely susceptible to ionizing radiation [40]. Thus, in addition to epithelial cell damage, some studies have suggested that capillary endothelial cell injury also plays a significant role in RIII. Indeed, blood in the stool is the most common reason for hospital visits in radiation proctitis patients, and these patients have telangiectatic bleeding as their main manifestation under endoscopy [41].

Radiation damages vascular endothelial cells in various ways, resulting in endothelial cell swelling, increased permeability, inflammatory cell adhesion and migration, and microthrombosis in microvessels. Pairs et al. [42] reported that endothelial apoptosis, rather than epithelial cell damage, was the primary lesion that triggered RIII in mice. They observed a clear instance of the apoptosis of intestinal vascular endothelial cells after 15 Gy irradiation in mice. Other researchers reported that protection against intestinal microcapillary endothelial cell apoptosis via COMP-Ang1, sphingosine-1-phosphate, and polydatin can alleviate RIII [43,44,45]. However, Schuller et al. [46] used different irradiation doses and irradiation methods to explore the role of endothelial cell apoptosis in RIII. They found that most apoptotic cells were located in the crypt epithelium, and there was no dose–response function of the vascular dose. Therefore, the role of endothelial cell apoptosis in RIII is controversial. It is now generally accepted that both epithelial and endothelial cells in the crypts are involved in RIII, with the epithelial cells being the most important target of radiation injury, which may subsequently induce apoptosis in endothelial cells [45].

In addition to apoptosis, vascular endothelial cells may also initiate cellular senescence after irradiation. The senescence of endothelial cells results in a senescence-related secretory phenotype in which endothelial cells secrete cytokines, proteins, and other factors that cause dysfunction in neighboring cells or contribute to a chronic inflammatory state [47].

Moreover, radiation increases the expression of integrins, selectins, vascular cell adhesion molecule 1 (VCAM-1), intercellular adhesion molecule 1 (ICAM-1), and platelet endothelial cell adhesion molecule-1 (PECAM-1) by the vascular endothelium, thereby changing the adhesion between endothelial cells and promoting the recruitment of macrophages, which affects vascular permeability and the local inflammatory response [47]. Studies have reported radiation-induced microvascular endothelial injury and thrombosis. Thrombomodulin is abundantly expressed in normal intestinal microvascular endothelial cells and is an important intravascular coagulation inhibitor that turns thrombin from procoagulation to anticoagulation. Radiation-induced reactive oxygen species upregulate TNF-α, IL-1, plasminogen activator inhibitor-1 (PAI-1), and other genes, which downregulate the expression of thrombomodulin, thereby promoting thrombus formation [48,49,50].

### 2.3. Gut Immune Imbalance

As the intestine is the largest immune organ in the body, it plays a vital role in the maintenance of immune homeostasis. The intestinal immune system, which recognizes and responds differently to the flora and antigens in the intestinal tract to maintain the immune balance of the intestinal tract and the whole body, comprises gut-associated lymphoid tissue (GALT) and secreted immunoglobulins [51]. The GALT of humans and mice comprises multi-follicular lymphoid tissues and numerous isolated lymphoid follicles (ILF), mainly including immune cells, such as T cells, B cells, dendritic cells (DC), mast cells, and macrophages [52,53]. Secretory immunoglobulin A (sIgA), a protein composed of two identical heavy (H) and light (L) chains connected by disulfide bonds, is the most highly secreted immunoglobulin in the gut [54]. The intestinal immune system plays a pivotal role in the maintenance of intestinal homeostasis, and its imbalance is closely related to intestinal diseases.

In RIII, the levels of immune cells and immune cytokines changed, suggesting an important role of immune imbalance in RIII. For example, Garg et al. [55] found that a single dose of 8.0 Gy gamma radiation in mice caused decreased numbers of neutrophils, macrophages, and B and T lymphocytes as early as 4 h after exposure. GATA-binding protein 3 (GATA-3) is thought to be the most important factor associated with the development of the Th2 phenotype and suppression of the Th1 phenotype [56]. In Wistar rats, fractionated radiotherapy induced elevated expressions of IL-1β, TNF-α, MCP-1, and iNOS in the distal colonic mucosa early after irradiation. At that time, the Th2-specific transcription factors GATA-3 and CCR4 promoted a Th2-like immune response [57]. Wang et al. [58] reported that radiation augmented the levels of immune-related factors INF-γ and TGF-β mRNA and decreased the levels of IL-17 mRNA, which indicates that ionizing radiation impairs intestinal immune function.

### 2.4. Gut Microbiome Imbalance

The gastrointestinal tract is rich in microbial flora that provides crucial benefits to hosts. Normal intestinal flora help form the intestinal biological barrier, which is one of the most important components of the intestinal mucosal barrier in addition to mechanical, chemical, and immune barriers. Dysbiosis is associated with the development of a variety of diseases, such as cancer, metabolic diseases, Alzheimer’s disease, and inflammatory bowel disease [59,60,61,62]. 

There is evidence that the pathophysiology of RIII is correlated with intestinal microbiota dysregulation. For example, Wang et al. [63] found dysbiosis in cervical cancer patients with RIII, characterized by significantly decreased α-diversity and increased β-diversity, a relatively higher abundance of Proteobacteria and Gammaproteobacteria, and a lower abundance of Bacteroides. In addition, Ferreira et al. [64] found that in prostate cancer patients with RIII, Phascolarctobacterium, Clostridium IV, and Roseburia were significantly increased. In animal models, Kim et al. [65] found that the levels of the genera Alistipes in the large intestine and genus Corynebacterium in the small intestine were increased, and the genera Prevotella in the large intestine and the genus Alistipes in the small intestine were decreased after irradiation treatment. Li et al. [66] found that localized irradiation resulted in a decreased ratio of Bacteroidetes to Firmicutes in mice. In conclusion, the present study shows that radiation can cause significant changes in the composition and diversity of the gut microbiota, mainly a decrease in probiotics and an increase in pathogens.

On the one hand, the imbalance of intestinal flora homeostasis disrupts the integrity of the intestinal epithelial barrier. Under normal physiological conditions, the gut microbiota prevents pathogen colonization through competition for shared nutrients and niches or commensal-mediated enhanced host defense mechanisms [67]. However, after radiation, decreased colonization by probiotics and increased colonization by pathogenic bacteria occurred, which led to the rearrangement and redistribution of the TJ proteins, a changed and disrupted mucus layer, and mucus barrier degradation [68]. Thus, the normal internal mechanical barrier and immune barrier are destroyed, and the permeability of the intestinal mucosa is increased, creating conditions promoting the migration of bacterial flora. On the other hand, the imbalance of intestinal flora homeostasis disrupts the normal immune balance of the body. Radiation causes the dysregulation of intestinal flora and metabolites, which influences the activation of immune cells, such as intestinal epithelial cells, dendritic cells, neutrophils, and Th17 cells, and the release of inflammatory cytokines, such as IL-6, IL-1β, and TNF-α, which ultimately aggravate the tissue damage [69,70,71].

## 3. Treatment Strategy of Radiation-Induced Intestinal Injury

**Figure 2:**
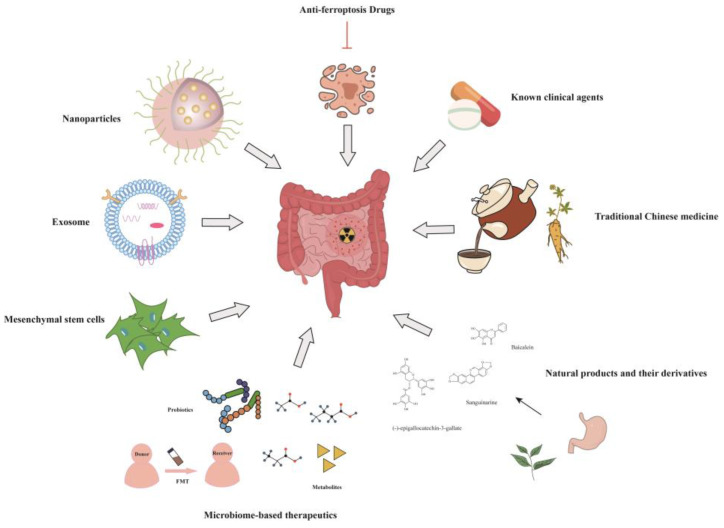
Potential treatment strategy of radiation-induced intestinal injury. Ionizing radiation causes intestinal damage. Various strategies, including known clinical agents, traditional Chinese medicine, natural products and their derivatives, microbiome-based therapeutics, mesenchymal stem cells (MSCs), exosomes, nanoparticles, and anti-ferroptosis drugs, have been demonstrated to ameliorate radiation-induced intestinal injury through a variety of mechanisms in preclinical and clinical trials.

To date, the clinical treatment of RIII is mostly symptomatic supportive treatment, and specific treatment strategies are lacking. For example, loperamide or compound phenoperidine is used to treat diarrhea, anticonvulsants are given to reduce colic, and antibiotics are used to inhibit the excessive proliferation of intestinal bacteria [3]. With the deepening of research on the mechanism of RIII, studies have shown that some novel drugs and therapeutic strategies have good therapeutic potential for RIII. In this section, we review potential therapeutic strategies for RIII based on preclinical and clinical trials (Figure 2).

### 3.1. Repurposing of Known In Vivo Therapeutic Agents 

Recent studies have shown that some known clinical agents have have corresponding therapeutic effects in animal or cell models of RIII (Table 1). For example, Bhanja et al. [72] found that BCN057, a small molecular antineoplastic agent, induces intestinal stem cell repair and mitigates RIII in vivo. They found that after lethal dose irradiation, the intestinal epithelial barrier of mice was destroyed, and the mortality rate was 100% within 15 days after radiation. Subcutaneous injection of BCN057 rescues ISCs, promotes intestinal epithelial regeneration, reduces intestinal injury, and improves the survival rate of irradiated mice.

Metformin (MF) is a widely used biguanide drug that is the first line of treatment for type 2 diabetes. In recent years, research has revealed its benefits in a variety of diseases, such as cancer, aging, and neurodegenerative diseases [73,74]. Jang et al. [24] investigated the role of MF in radiation-induced intestinal disease. They reported that MF increased the number of ISCs from radiation toxicity and enhanced epithelial repair by activating Wnt/β-catenin signaling in a mouse model. In addition, MF also protected mice from abdominal-radiation-induced intestinal damage by improving the composition and diversity of intestinal flora. The abundance of lactobacillus in the intestine of MF-treated patients was significantly increased during abdominal radiotherapy, which was negatively correlated with the duration of diarrhea [75]. 

Pravastatin (PS), which is widely used clinically to lower serum cholesterol levels, was found to improve endothelial and epithelial function after irradiation [76]. Jang et al. [77] found that PS can improve the RIII in mice and epithelial cell proliferation by inhibiting radiation-induced oxidative stress and inflammatory responses. Kwak et al. [78] evaluated the effect of PS in a minipig model and found that PS mitigated acute radiation enteropathy. They found that PS treatment upregulates the expression of metallothionein 2, thereby increasing the formation of the intercellular junction, enhancing epithelial integrity, and improving the intestinal epithelial barrier. 

Rebamipide is a gastrointestinal protective agent that is already used clinically to treat gastric ulcers and gastritis [79]. In a murine model of RIII, it promoted the recovery of intestinal barrier function and alleviated intestinal mucosal injury. Mechanistically, it can reduce inflammatory response by increasing intestinal cell proliferation and inhibiting the expression of MMP9 and pro-inflammatory cytokines. In addition, rebamipide can also significantly increase the number of goblet cells, accelerate the recovery of damaged tight junctions, and prevent bacterial translocation during acute radiation colitis [80]. 

Since oxidative stress is an important mechanism of radiation damage, N-acetylcysteine (NAC), a well-established antioxidant, is a potential therapeutic strategy for radiation damage. Mercantepe et al. [81] explored the protective effect of NAC against radiation-treatment-induced damage to the intestinal system. They divided rats into a control group, a radiotherapy group (RT), and an RT + NAC group. They found that malondialdehyde (MDA) levels and caspase-3 expression were increased and glutathione (GSH) levels were decreased in the RT group compared to the control group. Compared to the RT group, oral NAC significantly decreased the RT-induced MDA levels and caspase-3 expression in the small intestine and increased the GSH levels. Shukla et al. [39] evaluated the effects of NAC feeding on radiation-induced TJ and adherens junction (AJ) disruption and mucosal barrier dysfunction. They found that radiation rapidly damages TJ, AJ, and the actin cytoskeleton through an oxidative-stress-dependent mechanism, and feeding NAC before irradiation can block the radiation-induced damage of TJ, AJ, and the actin cytoskeleton and the increase in colonic mucosal permeability. These studies suggest that NAC may serve as a potential prophylactic treatment to prevent radiation-induced gastrointestinal mucositis and acute colon radiation syndrome.

**Table 1:** Repurposing of known in vivo therapeutic agents.

Therapy	Models	Functions	Potential Mechanism	References
BCN057	C57BL6/J mice	Intestinal epithelial regeneration	Induces intestinal stem cell repair	[72]
Metformin	Male C57BL/6 mice	Intestinal barrier	Wnt/β-catenin signaling	[24,73]
	Female BALB/c mice	Intestinal flora	Farnesoid X receptor (FXR)	[75]
Pravastatin	Male C57BL/6 mice; InEpC	Oxidative stress, and inflammatory response	IL-6, IL-1β, and TNF-α	[77]
	Minipigs; Male C57BL/6 mice	Intestinal epithelial barrier	Metallothionein 2	[78]
Rebamipide	Male C57BL/6 mice	Intestinal barrier	MMP9 and pro-inflammatory cytokines	[20,79]
		Intestinal barrier	goblet cell	[80]
N-acetylcysteine	Male Sprague Dawley rats	Villus and crypt epithelial cells	MDA, Caspase-3, GSH	[81]
	Female C57BL/6 mice	Mucosal barrier	TJ, AJ, and actin cytoskeleton	[39]

IL-6: interleukin-6; TNF-α: tumor necrosis factor α; MMP9: matrix metalloproteinase-9; MCP1:monocyte chemotactic protein-1; MDA: malondialdehyde; GSH: glutathione; TJ: tight junction; AJ: adherens junction.

### 3.2. Traditional Chinese medicine (TCM)

TCM refers to a holistic approach to diagnosis, pathophysiology, and treatment based on the accumulation of more than 2000 years of knowledge and practice, which includes herbal medicine, acupuncture, and other physical therapies [82]. Studies have shown that TCM has a good protective effect on RIII. Li et al. [83] established a radiation-induced intestinal edema (RIIE) model by irradiating SD rats with 6 Gy X-ray and evaluated the effect and underlying molecular mechanisms of Guiqi Baizhu Decoction (GQBZD) on RIIE. Radiation induced significant edema of the colon tissue in rats, resulting in the expressions of reactive oxygen species (ROS), HIF-1α, and AQP4, and decreased expression/activity of Na^+^/K^+^-ATPase. GQBZD can improve hypoxia and oxidative stress, regulate the expression of AQP4 and Na^+^/K^+^-ATPase, and has a protective effect on RIIE. Compound Kushen injection (CKI) is an injectable liquid made from the roots of Kushen (Radix sophorae flavescentis) and Baituling (Rhizoma smilacis glabrae). It has been used to treat solid tumors, inflammation, and other diseases [84]. In a radiation-induced gastrointestinal mucositis model in rats, CKI alleviated radiation-induced gastrointestinal injury by inhibiting mucosal inflammation and epithelial cell apoptosis. It can reduce villous epithelial damage, the number of apoptotic cells in the crypts, and the levels of intestinal mucosal inflammatory factors IL-1β and IL-6 [85]. Similarly, Shaoyao Decoction (SYD) also has a good protective effect on X-ray-induced enteritis in C57BL/6 mice. SYD treatment can reduce the levels of serum oxidation and proinflammatory cytokines (MDA, COX, IL-6, IL-1β, and TNF-α), promote the expression of pro-apoptotic proteins Bax, Caspase-3, and Cyto-c, and increase the level of anti-apoptotic protein B-cell lymphoma 2 (Bcl-2), thereby reducing the inflammation and apoptosis induced by radiation [86]. The abovementioned in vitro experiments show that TCM has a good protective effect against RIII.

### 3.3. Natural Products and Their Derivatives

Natural products are chemical substances with pharmacological or biological activity produced in nature by organisms such as plants, animals, insects, marine organisms, and microorganisms [87]. Studies have shown that these natural products and their derivatives play an important role in RIII (Table 2). Firstly, some natural products and their derivatives affect RIII by affecting the intestinal epithelial barrier. For example, TT-2, an active fraction of the rhizomes of Trillium tschonoskii Maxim (TT), can significantly improve the colony formation and proliferation abilities and inhibit the apoptosis of irradiated rat intestinal epithelial cell line 6 (IEC-6) cells. Oral TT-2 can significantly enhance the proliferation of intestinal crypt cells and promote the repair of intestinal epithelium after abdominal irradiation [88]. Similarly, ghrelin, an endogenous peptide hormone synthesized by ghrelin cells in the gastrointestinal tract, can also promote ISC self-renewal by activating Notch signaling, thereby improving intestinal dysfunction in irradiated mice [89]. Kiang et al. [22] found that ghrelin attenuates RIII through the NF-κB-AKT-MAPK signaling pathway network. Khayyal et al. [90] investigated the therapeutic effect of a specially formulated chamomile extract on radiation mucositis. They found that chamomile extract mitigated radiation-induced intestinal tissue inflammation, oxidative stress, and apoptosis by reducing the increase in TNF-α and myeloperoxidase (MPO) and apoptosis in irradiated mice, opening a new therapeutic approach for the treatment of radiation-induced intestinal mucositis. Sanguinarine (SAN) is a benzo [c] phenanthridine alkaloid extracted from Papaveraceae plants, which has antibacterial, anti-inflammatory, anti-tumor, and other biological activities. In a mouse model of RIII, SAN preconditioning mitigated intestinal damage and promoted intestinal recovery. SAN also suppressed the high-mobility group box 1 (HMGB1)/Toll-like receptor 4 (TLR4) pathway activation, thereby reducing the levels of inflammatory cytokines, such as IL-6, IL-8, TNF-α, and interferon γ (IFN-γ) [91]. Lu et al. [92] reported that 3,3′-diindolylmethane (DIM), a natural small molecule compound formed by the hydrolysis of indole-3-carbinol in gastric acid, can reduce intestinal radiation damage. DIM not only protected mice from lethality and weight loss caused by whole-abdominal irradiation (WAI) but also enhanced the WAI-induced reduction in Lgr5^+^ ISCs and their progeny cells, thereby promoting small intestinal repair after WAI exposure and improving small intestinal structural and functional damage. In addition, compared to WAI mice, the expression of nuclear factor erythroid 2-related factor 2 (Nrf2) was increased, the number of apoptotic cells and the expression of γ-H2AX was decreased, and the structure of the intestinal flora was changed in the small intestine after treatment with DIM. This suggests that DIM can improve intestinal oxidative stress, apoptosis, and DNA damage induced by radiation. (-)-Epigalloc atechin-3-gallate (EGCG) is a major polyphenol in green tea that has been studied for the treatment of a variety of diseases due to its powerful antioxidant activity. In RIII, it can prolong the survival time of lethally irradiated mice, reduce the intestinal mucosal damage caused by radiation, increase the number of Lgr5^+^ ISCs and their progenitor Ki67^+^ cells, and reduce the radiation-induced DNA damage and apoptosis. Mechanistically, it can protect RIII by clearing ROS and inhibiting apoptosis and ferroptosis through the Nrf2 signaling pathway. Similarly, Procyanidin B2 (PB2) is an oligomeric anthocyanidin precursor, which has a strong antioxidant effect [93]. PB2 can also inhibit oxidative stress and promote intestinal damage repair from irradiation through the Nrf2/ARE signaling pathway, and the knockdown of Nrf2 weakens the intestinal protection induced by PB2. More importantly, PB2 also promotes regeneration driven by Lgr5^+^ ISCs via Wnt/ β-catenin signaling. This indicates that PB2 is a promising new radioprotective agent [94]. 

Secondly, some natural products and their derivatives can regulate gut microbiota. Zheng et al. [94] found that after mice suffered whole-body irradiation (WBI), their survival rate and body weight were decreased, the expression of inflammatory factors in the serum was increased, and the gut microbiota was altered. The extract of fruits of Lycium barbarum (LBE) treatment can improve the survival rate and weight loss and inhibit the expression of inflammatory factors in mice induced by radiation. In addition, LBE treatment increased the relative abundance of potentially beneficial bacteria genera, such as Clostridium_sensu_stricto_1, Faecalibaculum, Akkermansia, and Turicibacter, and decreased the relative abundance of potentially harmful bacteria genera, such as Rikenellaceae_RC9_gut_group and Muribaculum. Baicalein is a plant-derived flavonoid. In a radiation-induced mouse intestinal injury model, baicalein improved the intestinal structure, proliferation, and regeneration of radiation-exposed mice, and the rebalancing of the intestinal microbial composition played an important role. Radiation increased the relative abundance of Bacteroides, Alistipes, Parabacteroides, and Ruminococcaceae_UCG-014, and decreased the relative abundance of Lactobacillus and Prevotellaceae_UCG-001. Subsequent KEGG results showed that Baicalin may promote the normalization of the intestinal microbial composition through p53-related apoptosis [95]. As mentioned above, Cai et al. investigated the role of EGCG in radiation-induced intestinal damage, and subsequently, they investigated the effects of EGCG on intestinal microbiota in irradiated mice [96]. They found that EGCG restored intestinal Firmicutes-to-Bacteroidetes (F/B) ratios, an indicator of intestinal inflammation and general physiological status, and increased the abundance of probiotics in irradiated mice. This study provides new insights into EGCG-mediated RIII remission.

Finally, natural products and their derivatives can affect endothelial cell function. Jang et al. [97] studied the therapeutic effect of baicalein on the endothelial dysfunction of intestinal inflammation induced by radiation. They found that baicalin significantly reduced the radiation-induced upregulation of adhesion molecules P-selectin, ICAM-1, and VCAM-1 and neutrophil infiltration, thereby promoting crypt regeneration and ameliorating radiation-induced damage in mice and cell models. This suggests that baicalin has a therapeutic effect on radiation-induced intestinal inflammation by alleviating endothelial damage. Paeoniflorin (PF) is a monoterpenoid glycoside, which has anti-inflammatory, hypoglycemic, and cognitive enhancement functions [98]. PF can reduce mortality and mucosal damage, reduce colon edema and inflammatory cell infiltration, and increase colon blood flow in radiation-exposed mice. Mechanically, PF may alleviate the damage of endothelial cells and macrophages by activating the growth-arrest-specific gene 6 (Gas6)/Axl/Suppressor of the cytokine signaling 3 (SOCS3) axis, thus effectively alleviating radiation enteritis [99]. These studies indicate that the natural products and their derivatives can affect the intestinal barrier by affecting the function of endothelial cells, which provides a new idea for the treatment of RIII.

**Table 2:** Natural products and their derivatives.

Therapy	Models	Functions	Potential Mechanism	Reference
TT-2	IEC-6; C57BL/6 mice	Repair of intestinal epithelium	Lgr5 ISC numbers	[88]
Ghrelin	Human intestinal epithelial cell line (InEpCs); Male C57BL/6 mice	Epithelial proliferation	Notch signaling	[89]
	Female B6D21 mice	Increases in epithelial proliferation and inhibition of pro-inflammatory cytokine production	A balance of NF-keppaB-AKT-MAPK network	[22]
Chamomile extract	Male Wistar rats	Tissue inflammation, oxidative stress, and apoptosis	TNF-α, MPO	[90]
Sanguinarine	Male C57BL/6J mice	Gut microbiota	HMGB1/TLR4	[91]
Diindolylmethane	Male C57BL/6J mice	Oxidative stress, apoptosis, and DNA damage	ROS, lysozyme Paneth cells, villin enterocytes, and Ki67	[92]
(-)-Epigalloc atechin-3-gallate	Male C57BL/6J mice; Human intestinal epithelial cell (HIEC)	Intestinal mucosal	Number of Lgr5^+^ ISCs and Ki67^+^ cells; Nrf2 signaling pathway	[6]
Procyanidin B2	Male Wistar rats	Oxidative stress and intestinal damage repair	Nrf2/ARE; Wnt/β-catenin	[93]
Land-based exercise	Male C57BL/6J mice	Gut microbiota	Inflammatory factors	[94]
Baicalein	Mice	Gut microbiota	P53	[95]
	Male C57BL/6J mice	Intestinal inflammation and intestinal barrier	P-selectin, ICAM-1, and VCAM-1	[97]
Paeoniflorin	Male C57BL/6J mice; RAW264.7 cells	Mucosal damage, colon edema and inflammatory cell	Gas6/Axl/SOCS3	[99]

IEC-6: intestinal epithelial cell line 6; MPO: myeloperoxidase; HMGB1: high mobility group box 1; TLR4: toll-like receptor 4; ROS: reactive oxygen species; PB2: procyanidin B2; Nrf2: nuclear factor erythroid 2-related factor 2; ARE: antioxidant response element; ICAM-1: intercellular adhesion molecule 1; VCAM-1: vascular cell adhesion molecule 1; Gas6: growth-arrest-specific gene 6; SOCS3: suppressor of cytokine signaling 3.

### 3.4. Microbiome-Based Therapeutics 

As the composition and quantity of intestinal flora are changed after irradiation and the composition of the microbiota is related to disease susceptibility, microbiome-based therapy has also become a research focus of RIII. 

Clinical and preclinical studies have shown that probiotics can alleviate RIII. VLS #3 is a mixture of eight different probiotic strains. Clinical studies have shown that VSL#3 can significantly reduce the incidence of diarrhea in patients with pelvic tumors undergoing radiation therapy [100]. A randomized, double-blind controlled trial evaluated the effect of Bifilact^®^ probiotics (Lactobacillus acidophilus LAC-361 and Bifidobacterium longum BB-536) on diarrhea during pelvic radiation therapy. A total of 246 patients were randomly assigned to the placebo or Bifilact^®^ probiotics groups. The study found that Bifilact^®^ probiotics significantly reduced radiation-induced grade 4 diarrhea compared to the placebo group [101]. In animal studies, antibiotics have also shown good therapeutic effects. Lactobacillus rhamnosus GG (LGG), a probiotic, has a radiation-protective effect on the intestinal tract of mice. Rihel et al. [102] showed that LGG releases radiation-protective lipoteichoic acid (LTA) and activates macrophage TLR2 and cyclooxygenase-2 (COX-2)-expressing mesenchymal stem cells (MSCs) to migrate to areas near epithelial stem cells, thereby protecting the intestinal epithelium from radiation- and chemo-induced damage. After rats suffered abdominal irradiation, supplementation with *L. casei* ATCC334 inhibited the growth of Escherichia/Shigella and favored the proliferation of Ackermannia. In addition, *L. casei* ATCC334 inhibited the production of putrescine and promoted the production of alpha-linolenic acid (ALA), which are significantly associated with the proliferation of ISCs and the enhancement of intestinal barrier function. Therefore, *L. casei* ATCC334 alleviates RIII by enhancing the mucosal barrier and remodeling the intestinal flora structure and metabolic activity [103]. Sittipo et al. [104] reported that the relative abundance of Lactobacillus decreased in mice exposed to a single dose of gamma rays of 6 Gy, especially at early time points after irradiation. Treatment with Lactobacillus acidophilus can promote the formation and budding of intestinal organoids, maintain stem cell stemness, and promote goblet cell recovery in irradiated mice. Zhou et al. [105] isolated a strain of Bacteroides fragilis from the feces of a healthy infant and named it *B. fragilis* strain ZY-312 (*B. fragilis*). *B. fragilis* plays a protective role in RIII by promoting IEC proliferation, stem cell regeneration, mucus secretion, and tight junction integrity through the upregulation of signal transducer and activator of transcription 3 (STAT3) signaling pathways. 

In addition to a single or multi-strain combined application, fecal microbiota transplantation (FMT) is also a new treatment strategy for RIII. In a mouse model, FMT can improve radiation-induced death and reduce radiation-induced hematopoietic injury, gastrointestinal function, and intestinal epithelial integrity. In addition, FMT reversed the downregulation of the relative abundance of Bacteroides (or Firmicutes) at the phylum level in mice caused by radiation exposure. This indicates that FMT may serve as a new therapeutic approach to improve patient outcomes after radiotherapy [106]. Indeed, a clinical trial supports this idea. In the study, five patients with chronic radiation enteritis (CRE) received FMT. The FMT treatment showed positive results in three patients, with improvements in diarrhea, rectal bleeding, abdominal/rectal pain, and fecal incontinence. This demonstrates the safety and efficacy of FMT in the treatment of patients with CRE [107]. 

The gut microbiota affects the host through the release of metabolites, which has also become one of the therapeutic strategies for RIII. Xiao et al. [108] found that FMT increased the content of microbiologically derived indole-3-propionic acid (IPA) in irradiated mice fecal pellets. Additionally, oral IPA improves radiation-induced hematopoiesis and intestinal damage, in which pregnancy X receptor (PXR)/Acyl-CoA binding protein (ACBP) signaling plays a key role. Among the gut microbiota, short-chain fatty acid (SCFA) is the topic of the most in-depth and extensive research at present. Valeric acid, a short-chain fatty acid, was shown to improve radiation-induced hematopoietic injury and restore gastrointestinal function and epithelial integrity through keratin 1 (KRT1) in mice [109]. Urolithin A (UroA) is a gut microbiota produced by foods containing ellagic acid, such as pomegranates, walnuts, and berries [110]. Uro A can improve intestinal survival, morphology, and function, reduce DNA damage and apoptosis, and restore the changes in the intestinal flora of mice after 9.0 Gy of irradiation. This indicates that UroA may be a potential target for radiation injury [111]. 

### 3.5. Mesenchymal Stem Cells (MSCs)

MSCs have multi-directional differentiation ability and can differentiate into a variety of cell lines, such as osteocytes, chondrocytes, adipocytes, and other lineages [112]. Therefore, MSCs play vital roles in tissue damage repair, including RIII. 

Sémont et al. [113] reported that the tail vein injection of MSCs can significantly improve radiation-induced gastrointestinal injury in mice, improve intestinal structure and function, and prolong the lifespan of mice. MSCs can restore cell homeostasis by improving intestinal epithelial renewal ability, promoting proliferation, and inhibiting apoptosis. Subsequent studies have found that the protective effect of MSCs is related to Lgr5^+^ ISCs. After irradiation, Lgr5^+^ ISCs and their daughter cells, such as Ki67^+^ TA cells, Vil1^+^ enterocytes cells, and lysozyme^+^ Paneth cells, were significantly increased under MSC treatment. This suggests that MSCs promote the growth of Lgr5^+^ ISCs, thereby promoting the repair of the small intestine after radiation exposure, which is associated with increased activation of the Wnt/β-catenin signaling pathway [114]. In addition, irradiation enhances the internalization of MSC-derived extracellular vehicles (Evs) in epithelial cells, and MSC-derived EV treatment can improve small intestinal epithelial renewal by stimulating the proliferation of epithelial crypt cells and inhibiting apoptosis [115]. Kim et al. [116] established a rat model of radiation-induced proctitis to investigate the therapeutic effects of human placenta-derived (PD) and adipose tissue-derived (AD) MSCs. They found that compared to the control group, both types of MSCs reduced rectal fibrosis, promoted mucosal proliferation, and inhibited apoptosis. An SD rat in vivo experiment investigated the ability of MSCs to reduce radiation-induced colorectal fibrosis. Studies have shown that MSCs reduce radiation-induced fibrosis by releasing hepatocyte growth factor (HGF) and TNF-stimulated gene 6 (TSG-6) to limit the activation of pro-fibrotic cells, particularly myofibroblasts, smooth muscle cells (SMCs), and macrophages [117]. These studies demonstrate the therapeutic effect of MSCs on RIII.

Moreover, some scientists have modified MSCs to achieve better targeting and therapeutic effects. For example, R-spondin1 (RSPO1), a cytokine containing the structural domain of platelet reaction, is a potent and specific epithelial mitogen that stimulates the growth of the productive intestinal mucosa. In a study, RSPO1 was transfected into MSCs to construct RSPO1-modified C3H10 T1/2 cells. The C3H10/RSPO1 cells could migrate to the site of injury, promote ISC survival, proliferation, and differentiation, and effectively repair radiation-induced intestinal epithelial cell injury [118]. HGF, a multifunctional cytokine involved in morphogenesis, cell survival, proliferation, and anti-inflammatory effects, promotes the regeneration of organs such as the intestine, liver, kidney, and lung [119]. HGF-modified MSCs can reduce the expression and secretion of inflammatory cytokines and increase the expression of anti-inflammatory cytokines, thereby alleviating local inflammation in RIII mice. In addition, it can promote the proliferation of intestinal epithelial cells, reduce apoptosis, and promote the recovery of intestinal histopathology. This might provide an effective therapeutic strategy for RIII [120]. Chang et al. [121] overexpressed the mouse chemokine C-X-C motif chemokine 12 (CXCL12) gene in human-adipose-derived MSCs to treat RIII. They found that CXCL12-modified MSCs can promote intestinal epithelial repair in irradiated mice and are better than unmodified cells for enhancing the host’s repair response to radiation stress. This indicates that CXCL12-modified MSCs confer radioresistance to the intestinal epithelium. Ginsenoside RG1 (RG1) partially enhances the paracrine function of BM-MSCs by upregulating HO-1. BM-MSCs pre-activated by RG-1 (RG1-MSC-CM), rather than MSC-CM, significantly improved the survival and intestinal damage in irradiated rats by improving intestinal proliferation/apoptosis, inflammation, angiogenesis, and stem cell regeneration via HO-1-dependent mechanisms. This indicates that RG1 enhances the paracrine effect of BM-MSCs on RIII [122].

In addition to the exogenous administration of MSC therapy, drug stimulation of endogenous MSC migration to the injured area has also become a therapeutic strategy. As mentioned above, LGG mediates radiation protection by promoting the wound repair process through the release of LTA to promote the activation of TLR2 in macrophages and the migration of COX-2 from MSCs to areas near epithelial stem cells [102]. Photobiomodulation (PBM) accelerates wound healing via stimulating angiogenesis, in which stem cells are particularly susceptible to the effects of PBM. PBM enhances the angiogenic potential of MSCs, thereby improving the therapeutic efficacy in the treatment of radiation-induced enteropathy [123]. 

### 3.6. Exosome 

Exosomes are produced by various cell types, serve as mediators of intercellular communication, and influence biological functions by transferring microRNAs (miRNAs), long non-coding RNAs (lncRNAs), proteins, and lipids [124]. Studies have shown that exosomes are involved in the process of RIII. Exosomes isolated from mouse serum after 24 h of TBI (Exo-IR-24h) reduced the overall survival of mice after 9 Gy TBI. Treatment with Exo-IR-24h reduced the number of cupped cells in the small intestine and enhanced TBI-induced small intestinal injury in mice. This injury can be rescued by GW4869, an inhibitor of exosome biogenesis and release [125], which suggests that exosomes are involved in RIII and that exosomes may be a potential therapeutic target. Indeed, Yang et al. [126] investigated the role of MSC-derived exosomes (MSC-exos) in RIII. They found that the injection of MSC-exos suppressed the inflammatory response, increased the expression of stem cell markers, and maintained the integrity of the intestinal epithelium in irradiated mice. Mechanistically, MSC-exos promote the proliferation and differentiation of Lgr5^+^ intestinal epithelial cells through the miR-195/Akt/β-Catenin signaling pathway, which provides a theoretical basis for exosomes in the treatment of RIII. However, there are few studies in this area, and the effect of exosomes needs further research.

### 3.7. Nanoparticles

Nanomedicine combines nanoparticles with therapeutic agents to improve drug targeting, enhance drug concentration, and achieve in vivo tracer and other effects. Therefore, nanoparticle therapy is used in various fields of medicine [127]. Researchers have constructed a series of nanoparticles for the prevention and treatment of RIII (Table 3). For example, Wang et al. [128] investigated the intestinal protective effect of the first clinically approved carbon nanoparticle suspension injection (CNSI) in China against RIII. They found that CNSI has high ROS scavenging activity, and the oral administration of CNSI can effectively inhibit the apoptosis of small intestinal epithelial and crypt stem cells, reduce the damage to the mechanical barrier, maintain the balance of the intestinal flora, and thus alleviate RIII. This suggests new applications of clinically approved carbon nanoparticles. Jia et al. [129] developed polydopamine nanoparticles (PDA-NPs) by binding dopamine hydrochloride to nanoparticles. The nanoparticle is spherical with good colloidal dispersion stability and accurate quantification. It can significantly reduce the mortality, prolong the life span, and reduce the intestinal injury of IR mice. As mentioned above, exosomes can be used as a potential therapeutic strategy for RIII, and exosome-coated nanoparticles have also been synthesized. The nanoparticles exo-PD are easily endocytosed by the cells in the body and accumulate in the organs, and the action time in the intestine is longer. Exo-PD can improve mitochondrial function through the PI3K-AKT pathway and alleviate RIII [126]. CeO_2_/Mn_3_O_4_ nanocrystals are metallic nanomaterials. They show better catalytic activity than CeO_2_ or Mn_3_O_4_ alone and are highly catalytic antioxidants. Only a small dose of CeO_2_/Mn_3_O_4_ nanocrystals was needed to protect intestinal organoids from radiation-induced injury and improve the survival rate of irradiated mice [130]. The abovementioned studies show that nanoparticles have strong targeting and stability and can enhance the function of drugs, which provides a reference for the clinical treatment of RIII by nanoparticles.

### 3.8. Anti-Ferroptosis Drug

As mentioned above, regarding the study of RIII, researchers have conducted more in-depth research on the mechanism of RIII, and it has been found that ferroptosis may affect the course of RIII, so strategies targeting ferroptosis may also become novel therapeutic strategies for RIII (Table 3). 

Ferrostatin 1 (Ferr-1) is a potent lipid peroxidation inhibitor that inhibits changes associated with ferroptosis [131]. In IEC-6 cells, Ferr-1 protected IEC-6 cells by reducing ROS production and inhibiting ferroptosis and p53-mediated apoptosis signaling pathways. In TBI mice, Ferr-1 increased survival and restored intestinal structure and physiological function after irradiation [33,132]. Liproxstatin 1 (Lip-1), a spiroquinoxalide derivative, also effectively and specifically inhibited ferroptosis [133]. Lip-1 treatment reduced lipid peroxidation and the expression of ferroptosis-associated genes and inflammatory factors, thereby improving intestinal damage in irradiated mice. Lip-1 can promote the recovery of intestinal immune function after exposure and may inhibit ferroptosis after radiation exposure by regulating the lysophosphatidylcholine acyltransferase 3 (LPCAT3)/lysyl oxidase (LOX) pathway [58]. Deferoxamine (DFO), an iron chelator, also reverses radiation-induced ferroptosis. At the in vitro level, DFO pretreatment decreased irradiation-induced ferroptosis and increased the survival rate of HIEC cells. At the in vivo level, DFO pretreatment significantly inhibited irradiation-induced iron accumulation in the intestinal tract, improved the survival rate, and alleviated intestinal tissue damage in mice [134]. Acyl-CoA Synthetase Long-Chain Family Member 4 (ACSL4) is an important component of the execution of ferroptosis, and it is significantly upregulated in intestinal tissues after IR. Intraperitoneally injecting mice with troglitazone, an ACSL4 inhibitor, significantly reduced tissue damage, lipid peroxidation, and ferroptosis [135]. Amifostine has always been the only small-molecule radioprotective agent approved by the FDA, and in order to address its toxicity and maintain its good potency, Zhang et al. [136] prepared a series of modified small polycysteine peptides. Among them, compound 5 could improve the hematopoietic system, lung tissue, and gastrointestinal syndrome caused by WBI. Additionally, compound 5 ameliorated RIII by regulating redox balance and amino acid anabolism and inhibiting ferroptosis. This indicates that compound 5 is a new type of radiation-protective agent. The abovementioned studies show that anti-ferroptosis drugs have been shown to improve RIII in both in vivo and in vitro experiments, which may be a potential therapeutic target for RIII.

**Table 3:** Nanoparticles and anti-ferroptosis drugs.

Therapy	Models	Functions	Potential Mechanism	Reference
Carbon nanoparticle suspension injection	IEC-; BALB/c male mice	Mechanical barrier; intestinal flora	ROS	[128]
Polydopamine nanoparticles	Male C57BL/6J mice; HIEC	Intestinal barrier function	ZO-1; Lgr5 ISCs	[129]
Exosomal programmed cell-death	C57BL/6J mice; HIEC-6; hAFSc	Mitochondrial function	PI3K-AKT	[126]
CeO_2_/Mn_3_O_4_ nanocrystals	Mice	Radiation-induced injury	ROS	[130]
Liproxstatin-1	Male Balb/c mice	Intestinal immune	LPCAT3/LOX	[58]
Deferoxamine	HIEC	Iron accumulation;	Prostaglandin-endoperoxide synthase 2 (PTGS2), GPX4, GSH	[134]
Acyl-CoA Synthetase Long-Chain Family Member 4	Female C57BL/6 mice	Tissue damage, lipid peroxidation, and ferroptosis		[135]
Amifostine	Male C57BL/6 mice; BALB/c-Nude mice	Redox balance, amino acid anabolism, and inhibiting ferroptosis	GPX4, GSH	[136]

HIEC: human intestinal epithelial cell; ZO-1: zona occludens 1; hAFSc: human amniotic fluid stem cells; PI3K-AKT: phosphatidylinositol 3-kinase-AKT; LPCAT3: lysophosphatidylcholine acyltransferase 3; LOX: lysyl oxidase; DFO: deferoxamine; GPX4: glutathione peroxidase 4; GSH: glutathione.

### 3.9. Combined Therapy

Some drugs may not produce significant results when used as a single therapy, but combination therapy can significantly improve the effectiveness.

In animal experiments, Kim et al. [137] used a combination of PS and MF in a minipig model and found that the combined treatment could attenuate radiation-induced intestinal histological damage, significantly increase the survival rate of the lethal dose radiation model, and achieve better therapeutic effects. Najafi et al. [138] investigated the possible radioprotection of the ileum and colon in rats using a combination of melatonin and metformin. They found that the combination of melatonin and metformin could completely prevent radiation toxicity and could serve as an ideal radioprotective agent for the colon. Zhang et al. [139] found that a combination of glucagon-like peptide-2 (GLP-2) and glutamine (Gln) significantly reduced radiation-induced enteric epithelium apoptosis. Studies have shown that a combination of pegylated G-CSF (a cytokine that promotes neutrophil maturation and mobilization) and Alxn4100TPO (a thrombopoietin receptor agonist) can significantly prolong the survival rate, reduce weight loss, and improve mitigate ileum histopathology of mice after WBI alone and followed by wound trauma [140].

In clinical trials, Wedlake et al. [141] grouped 308 pelvic radiotherapy patients for drug treatment using statins and ACE inhibitors (ACEi) either alone or in combination. The effectiveness was evaluated using the Inflammatory Bowel Disease Questionnaire-Bowel (IBDQ-B) score. The study found that both statins and the combination with ACEi resulted in a significant decrease in IBDQ-B scores, indicating that the combined use of drugs during pelvic radical radiotherapy can significantly reduce intestinal damage.

## 4. Discussion

As there are currently no FDA-approved drugs for the treatment of RIII, it is urgent to study the mechanisms and treatment strategies of RIII. This article reviewed the pathogenesis mechanism and potential therapeutic strategies of RIII based on preclinical studies to provide new ideas for the diagnosis and treatment of RIII.

This paper reviewed the damage mechanism of RIII based on the intestinal barrier, which provides a basis for the treatment of RIII. Radiation acts on the intestine and can cause damage to a variety of intestinal cells. At present, the mechanism of cell injury is mainly the apoptosis and necrosis of epithelial cells and endothelial cells. This review also summarized the role of ferroptosis in RIII. Ferroptosis is a regulated form of cell death driven by phospholipid peroxidation [142]. It is now believed that ferroptosis is probably one of the most prevalent and oldest forms of cell death. Current studies have shown that ferroptosis is associated with a variety of diseases and can be used as a therapeutic target for a variety of diseases [143,144]. Ferroptosis also plays an important role in promoting RIII, which provides a new direction for the study of the injury mechanism and treatment of RIII.

We also reviewed potential treatment strategies for RIII based on preclinical studies. The safety of known in vivo therapeutic drugs and TCM has been verified in other diseases, and the research on these drugs is conducive to shortening the drug development cycle. Other potential therapeutic agents provide a basis for the clinical treatment of RIII. For example, the microbiome, especially the gut microbiome, shares a broad interface with the host immune system and is closely related to the occurrence and development of many diseases [145]. Therefore, microbiome-based therapeutics is a promising medical treatment. Due to its targeting and high efficiency and stability, nanomedicine has shown good application prospects in the treatment of diseases. In this review, we also found that some nanoparticles showed good therapeutic effects on radiation-induced animal and cell damage, so nanoparticles are also a promising approach for the treatment of RIII. As the human body is a complex whole, multiple molecules are involved in the pathogenesis of disease, and the complexity of a given disease limits the efficacy of a single drug treatment. The combination therapy has a synergistic effect, can reduce the dosage of drugs and side effects, and has been proven to have better therapeutic effects in many diseases [146]. Our paper also revealed that combination therapy improves the survival rate and significantly improves radiation-induced enterotoxicity. Therefore, combination therapy may also be one of the future strategies for the treatment of RIII.

In addition, more precise radiation therapy can reduce the incidence of radiation damage. For example, stereotactic body radiation therapy (SBRT) is a relatively novel approach to irradiation that delivers higher doses of radiation to the tumor in only a few treatment fractions [147] and has been used to treat a variety of cancers. In the treatment of pancreatic cancer, SBRT can safely facilitate marginal negative resection in borderline resectable pancreatic cancer (BRPC) while avoiding damage to normal tissues [148,149]. SBRT also demonstrates significant activity against hepatocellular carcinoma (HCC), with a one-year local control rate of 87% [150]. Additionally, SBRT also shows certain efficacy in bile duct cancer. SBRT combined with immunotherapy has a higher potential to activate immune responses in the tumor region. After combined treatment, the survival time exceeded 26 months, showing good prospects [151]. Moreover, SBRT has also been used in abdominal and pelvic tumor therapy. For example, in the treatment of abdominal/pelvic lymph nodes, although radiotherapy may be a treatment option for salvage therapy, the treatment is very difficult due to the random movement of abdominal/pelvic lymph nodes under the influence of breathing and their location close to the gastrointestinal tract [152]. SBRT is a rapid, safe, and effective treatment option with a low risk of treatment-related toxicity for the treatment of single or multiple abdominal and pelvic lymph nodes [153]. Therefore, more accurate radiation therapy is essential for reducing the incidence of RIII.

In summary, this review summarized the damage mechanisms and potential therapeutic strategies for RIII. As there are currently no FDA-approved drugs for the treatment of RIII, these potential therapeutic strategies may provide new directions for the clinical treatment of RIII.

## Data Availability

Not applicable.
